# Dual‐Depletion of Intratumoral Lactate and ATP with Radicals Generation for Cascade Metabolic‐Chemodynamic Therapy

**DOI:** 10.1002/advs.202102595

**Published:** 2021-10-29

**Authors:** Feng Tian, Shiyao Wang, Keda Shi, Xingjian Zhong, Yutian Gu, Yadi Fan, Yu Zhang, Mo Yang

**Affiliations:** ^1^ Department of Biomedical Engineering The Hong Kong Polytechnic University Kowloon Hong Kong SAR China; ^2^ Department of Lung Transplant The First Affiliated Hospital School of Medicine Zhejiang University Hangzhou Zhejiang Province 310027 China; ^3^ Department of Mechanical and Automotive Engineering Royal Melbourne Institute of Technology University Melbourne Victoria 3000 Australia

**Keywords:** adenosine triphosphate, dual‐depletion, lactate, synergistic cancer therapy, tumor metabolism

## Abstract

Increasing evidence has demonstrated that lactate and adenosine triphosphate (ATP) both play important roles in regulating abnormal metabolism in the tumor microenvironment. Herein, an O_2_ self‐supplying catalytic nanoagent, based on tannic acid (TA)–Fe(III) coordination complexes‐coated perfluorooctyl bromide (PFOB) nanodroplets with lactate oxidases (LOX) loading (PFOB@TA–Fe(III)–LOX, PTFL), is designed for cascade metabolic‐chemodynamic therapy (CDT) by dual‐depletion of lactate and ATP with hydroxyl ^•^OH radicals generation. Benefiting from the catalytic property of loaded LOX and O_2_ self‐supplying of PFOB nanodroplets, PTFL nanoparticles (NPs) efficiently deplete tumoral lactate for down‐regulation of vascular endothelial growth factor expression and supplement the insufficient endogenous H_2_O_2_ . Simultaneously, TA–Fe(III) complexes release Fe(III) ions and TA in response to intracellular up‐regulated ATP in tumor cells followed by TA‐mediated Fe(III)/Fe(II) conversion, leading to the depletion of energy source ATP and the generation of cytotoxic ^•^OH radicals from H_2_O_2_. Moreover, TA–Fe(III) complexes provide photoacoustic contrast as imaging guidance to enhance therapeutic accuracy. As a result, PTFL NPs efficiently accumulate in tumors for suppression of tumor growth and show evidence of anti‐angiogenesis and anti‐metastasis effects. This multifunctional nanoagent may provide new insight for targeting abnormal tumor metabolism with the combination of CDT to achieve a synergistic therapeutic effect.

## Graphical abstract

This paper reports an O_2_ self‐supplying theranostic nanoagent (PFOB@TA–Fe(III)–LOX, PTFL nanoparticles) with cascade catalytic reaction for synergistic metabolic‐chemodynamic therapy by dual‐depletion of lactate and adenosine triphosphate together with the conversion of H_2_O_2_ to ^•^OH radicals. Results demonstrate that such nanoagent efficiently accumulates at the tumor sites for anti‐angiogenesis, anti‐metastasis, and anti‐tumor growth.

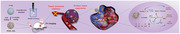

## 1 Introduction

Regulating the tumor microenvironment (TME) has been considered as an emerging effective strategy for cancer therapy.^[^ ^1^, ^2^ ^]^ The abnormal metabolism of cancer cells gives TME unique features including low pH,^[^ ^3^ ^]^ hypoxia,^[^ ^4^, ^5^ ^]^ redox imbalance,^[^ ^6^, ^7^ ^]^ and enzyme overexpression.^[^ ^8^ ^]^ One feature of tumor metabolism is the Warburg effect, that is, cancer cells massively convert glucose into lactate even with adequate oxygen (O_2_), leading to local lactate accumulation.^[^ ^9^, ^10^ ^]^ In the past decades, lactate was regarded as a metabolic waste product of glycolysis in tumor cells, which disrupted the acid‐base balance of tumor.^[^ ^11^ ^]^ However, increasing evidence recently has manifested that lactate accumulation leads to tumor angiogenesis, metastasis, and immune suppression to promote tumor growth and invasion.^[^ ^11^, ^12^, ^13^, ^14^, ^15^ ^]^ Hence, lactate is believed to be an effective target to regulate abnormal metabolism in TME by blocking generation or direct depletion.^[^ ^16^, ^17^, ^18^, ^19^, ^20^ ^]^ Lactate oxidase (LOX) is a natural bio‐enzyme for efficient lactate depletion. In the presence of O_2_, LOX catalyzes lactate to pyruvate and hydrogen peroxide (H_2_O_2_).^[^ ^21^, ^22^ ^]^ Moreover, compared to other metabolic substrates such as glucose,^[^ ^23^ ^]^ lactate level is extremely high in tumors and low in normal tissues (Table S1, Supporting Information).^[^ ^24^, ^25^, ^26^ ^]^ Hence, lactate depletion based on LOX has minimal side effects compared to glucose depletion based on glucose oxidase.^[^ ^27^, ^28^ ^]^ Unfortunately, there are only a few studies that have integrated LOX into nanoplatforms to regulate lactate levels for cancer therapy, and the tumor metabolic therapeutic effect of LOX‐based nanoplatforms is limited only by lactate depletion.^[^ ^18^, ^19^ ^]^ Moreover, the therapeutic effect based on LOX is notably hindered by the lack of O_2_ in TME. Although the by‐product H_2_O_2_, a type of reactive oxygen species (ROS), can further cause cell death, the effect is not good enough due to the relatively weak reactivity among all types of ROS.^[^ ^29^ ^]^ Hence, additional elements are in demand to synergistically fill the gap and enhance the therapeutic efficacy, leading to a win‐win outcome.

Adenosine triphosphate (ATP) is another main biochemical component in TME to provide energy for tumor cell survival and proliferation.^[^ ^30^, ^31^ ^]^ Due to excessive glycolysis, ATP is upregulated and highly concentrated in tumor cells (1–10 × 10^−3^ m). Hence, ATP depletion has been considered as one promising approach to inhibit tumor growth,^[^ ^32^, ^33^ ^]^ and combining ATP depletion with lactate depletion may further enhance tumor metabolic therapy effect. Perfluorocarbon (PFC), with chemical inertness and excellent biocompatibility, is highly active in medical fields such as artificial blood,^[^ ^34^ ^]^ organ transplantation,^[^ ^35^ ^]^ and cancer treatment^[^ ^36^, ^37^ ^]^ due to its high O_2_ solubility. For cancer treatment, PFC‐based nanosystems can supply O_2_ to relieve the hypoxia in TME for O_2_‐demand therapies.^[^ ^36^, ^37^ ^]^ Chemodynamic therapy (CDT) is now widely used for its unique way to generate ROS. With the conversion from H_2_O_2_ to hydroxyl radical (^•^OH) by Fenton/Fenton‐like reactions, CDT can more efficiently induce cell apoptosis and necrosis.^[^ ^32^, ^37^, ^38^, ^39^, ^41^ ^]^ However, CDT is generally limited by insufficient endogenous H_2_O_2_. It is reported that the chelate tannic acid (TA) and iron ions (TA–Fe(III)) complexes could respond to ATP and effectively induce the conversion from H_2_O_2_ to ^•^OH.^[^ ^33^, ^42^, ^43^ ^]^ Therefore, if PFC and ATP‐responsive CDT agents are integrated with LOX, a cascade catalytic route is formed. The O_2_‐sufficient PFC supplies O_2_ to promote the lactate depletion for anti‐angiogenesis/metastasis and generate H_2_O_2_. Simultaneously, the ATP‐responsive CDT agent consumes ATP and converts the produced H_2_O_2_ to cytotoxic ^•^OH radicals without consuming additional O_2_ to further suppress the tumor growth.

**Scheme 1:**
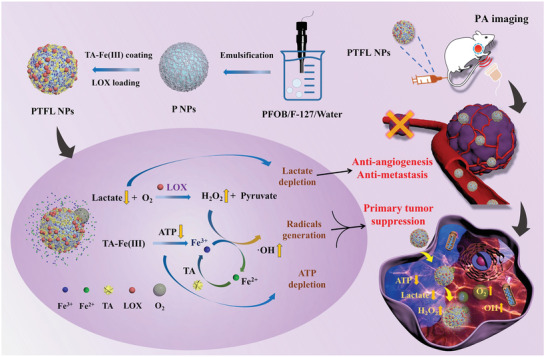
a) Schematic illustration of PTFL NPs synthesis. b,c) Schematic illustration of PTFL NPs for PA imaging‐guided synergistic and cascade metabolic‐chemodynamic therapy.

Herein, for the first time, an O_2_ self‐supplying theranostic nanoagent with cascade catalytic reaction is designed for synergistic metabolic‐CDT by dual‐depletion of lactate and ATP together with the conversion of H_2_O_2_ to ^•^OH radicals. As shown in **Scheme** **1**, this theranostic nanoagent consists of a perfluorooctyl bromide (PFOB) nanodroplet core coated with a LOX‐loaded TA–Fe(III) complex layer (PFOB@TA–Fe(III)–LOX, PTFL). Here, TA‐Fe(III) complexes coating can stabilize PFOB nanodroplets and also load LOX molecules. After being internalized by tumor cells, i) the O_2_ self‐sufficient PTFL nanoparticles (NPs) deliver O_2_ to relieve hypoxia. The loaded LOX catalyzes the oxidation reaction of lactate with O_2_, leading to lactate depletion and the generation of H_2_O_2_.^[^ ^21^, ^22^ ^]^ As a result, lactate depletion regulates abnormal metabolism in tumors and down‐regulate vascular endothelial growth factor (VEGF)/cluster of differentiation 31 (CD31) expression to promote anti‐angiogenesis.^[^ ^14^, ^15^, ^18^, ^19^ ^]^ ii) At the same time, TA–Fe(III) complexes exhaust intracellular ATP as an energy source and decompose to TA and Fe (III) ions. In this way, ATP was depleted to inhibit tumor growth.^[^ ^33^ ^]^ iii) Moreover, Fe(III) ions released during ATP depletion are further reduced into Fe(II) ions by TA and subsequently catalyze H_2_O_2_ generated during lactate depletion to cytotoxic ^•^OH, thereby enhancing CDT (Scheme 1).^[^ ^42^, ^43^ ^]^ Last, TA–Fe(III) complexes also provide photoacoustic (PA) imaging contrast as imaging guidance to further enhance the total therapeutic accuracy and efficacy.^[^ ^44^ ^]^ This lactate/ATP dual‐depletion catalytic nanoagent could effectively suppress tumor growth and invasion by a cascade tumor metabolic‐CDT, which might provide new insight for abnormal metabolism‐targeted cancer therapy with the combination of CDT to achieve a synergistic therapeutic effect.

## 2 Results and Discussion

### 2.1 Design, Fabrication, and Characterization of PTFL NPs

PTFL NPs are synthesized based on the steps shown in Scheme 1a. First, PFOB in pluronic F‐127 (F‐127) aqueous solution is emulsified under probe sonication to obtain PFOB NPs (P NPs). As a biocompatible surfactant, F‐127 polymers wrap around the surface of PFOB nanodroplets and act as stabilizers. Then, iron chloride, LOX, and TA are added to form the PFOB@TA–Fe(III)–LOX (PTFL) complex. LOX, as a protein, is amphiphilic in nature, which can help F‐127 stabilize the PFOB nanodroplets, thereby attaching to the surface of the PFOB nanodroplets via hydrophobic interaction. In addition, Fe(III) ions can coordinate with the ethylene oxide of the F‐127 polymer and can also coordinate with LOX through amino acid residues on the surface of the protein, thereby acting as a bridge for loading more LOX from the solution.^[^ ^43^, ^45^ ^]^ After the addition of TA, TA forms hydrogen bonds with F‐127 and LOX, and coordinates with Fe(III) ions together to form a final complex layer (F127–Fe(III)–LOX–TA) because of the abundant dihydroxyphenyl and trihydroxyphenyl groups.^[^ ^43^, ^46^ ^]^ Finally, the formed complex layer encapsulates PFOB nanodroplets and also loads LOX into NPs. Here, PFOB NPs loaded with LOX only, TA–Fe(III) complex only, and LOX+TA–Fe(III) are abbreviated as PL NPs, PTF NPs, and PTFL NPs, respectively.

**Figure 1:**
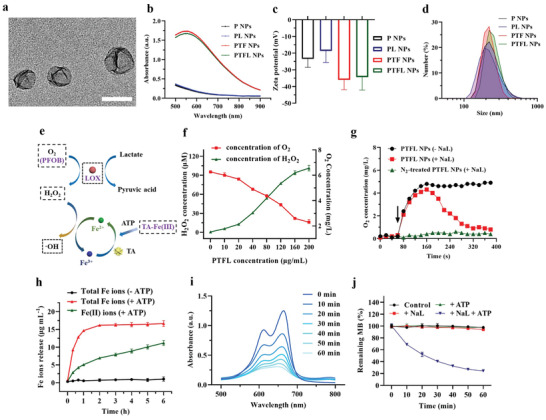
a) TEM image of PTFL NPs. Scale bar: 200 nm. b–d) Vis‐NIR extinction, Zeta potential, and DLS of P NPs, PL NPs, PTF NPs, and PTFL NPs. Data in (c) are shown as *n* = 3, mean ± SD. e) Schematic illustration of PTFL NPs induced H_2_O_2_ and ^•^OH generation. f) Concentration of H_2_O_2_ and dissolved O_2_ generating from 15 min reaction between various concentrations of PTFL NPs and 10 mm NaL at room temperature (*n* = 3, mean ± SD). g) O_2_ concentration after adding PTFL NPs to N_2_‐treated solution (with/without 10 mm NaL). h). The time‐dependent concentration of total Fe ions release and the converted Fe^2+^ ions from PTFL NPs by 1 mg mL^−1^ ATP or without ATP addition, respectively. (*n* = 3, mean ± SD). i) Time‐dependent methylene blue (MB) (10 mm) degradation by addition of PTFL NPs (200 µg mL^−1^), ATP (1 mg mL^−1^), and NaL (10 mm). j) The ratio of remaining MB after treated with 1) 1 mg mL^−1^ ATP, 2) 10 mm NaL, 3) 1 mg mL^−1^ ATP + 10 mm NaL. MB without any treatment was set as control (*n* = 3, mean ± SD).

Transmission electron microscopy (TEM) image showed that the synthesized PTFL NPs had a core‐shell structure and were monodispersed in the water with an average size of 182 ± 13 nm (**Figure** **1**a). Energy dispersive spectroscopy (EDS) elemental mapping also indicated the co‐existing of O element from F127 and TA and Fe element from coordinated iron ions in PTFL NPs (Figure S1, Supporting Information). Vis–NIR extinction spectra manifested the PTFL NPs had the extinction capability at the NIR region (Figure 1b) due to the coating of TA–Fe(III) complexes. Zeta potential measurements indicated the P NPs were −23.4 mV (Figure 1c) due to the negatively charged surface of PFOB cores.^[^ ^47^, ^48^ ^]^ The loading of LOX on P NPs weakened the zeta potential to −18.7 mV, while the formation of PTFL NPs with TA‐Fe(III) complex and LOX coating led to a zeta potential of −34.4 mV (Figure 1c). The highly negatively charged surface of PTFL NPs ensured the good water dispersity. To explore the effect of the order of adding TA or Fe(III) first together with LOX on the loading capacity of LOX, the LOX loading capacities of these two cases were measured. The results revealed that the loading capacity of LOX in the case to add TA first was ≈0.7%, and the loading capacity was ≈1% when Fe(III) was added first. These results indicated that adding Fe(III) together with LOX first to form PFOB@Fe(III)–LOX complex and then followed by adding TA for chelation with Fe(III) could increase the LOX loading capacity. This could be attributed to the weakening of electrostatic repulsion between PFOB and LOX by the positive charge of iron cations during LOX loading. The zeta potential of PTFL NPs of adding TA first (‐ 34.3 mV) was lower than that of PTFL NPs of adding Fe(III) first (−32.5 mV), indicating a higher loading of weak negatively charged LOX on PTFL NPs by adding Fe(III) first (Figure S2, Supporting Information).

Dynamic light scattering (DLS) measurement indicated that the dynamic sizes of P NPs, PL NPs, PTF NPs, and PTFL NPs were around 260, 256, 253, and 257 nm, respectively, but the polydispersity indices of P NPs and PL NPs were larger (Figure 1d). This could be explained that the coating of TA–Fe(III) complexes stabilized PFOB cores and helped the NP well dispersed. To further confirm this, DLS sizes of P NPs, PL NPs, PTF NPs, and PTFL NPs were measured at 1, 2, 4, and 7 days after synthesis. Results showed that the sizes of P NPs and PL NPs increased to 412 and 406 nm after 7 days, whereas the size of PTF NPs and PTFL NPs showed no obvious difference (Figure S3, Supporting Information). The results demonstrated that the coating of TA–Fe(III) complexes played a stabilizing role. In addition, after dispersing in water, phosphate buffered saline (PBS) (pH = 7.4 or 5.5), or cell culture medium for 48 h, no obvious differences in the appearance and DLS sizes of PTFL NPs were observed (Figure S4, Supporting Information).

### 2.2 PTFL NPs Catalyzed Lactate Depletion, ATP Triggered Fe Ions Release, and ^•^OH Generation

As mentioned above, LOX can catalyze the decomposition of lactate in the presence of O_2_ and generate pyruvate and H_2_O_2_ (Figure 1e). Hence, O_2_ and H_2_O_2_ levels were selected as indicators of the catalytic activity of PTFL NPs. H_2_O_2_ concentration was measured using an H_2_O_2_ assay kit based on an H_2_O_2_ standard curve (Figure S5, Supporting Information), while the level of O_2_ was determined by a portable dissolved O_2_ meter. Different concentrations of PTFL NPs (0, 10, 20, 40, 80, 120, 160, 200 µg mL^−1^) were mixed with sodium lactate (NaL) without the addition of ATP to avoid the needless release of iron ions or generation of radicals which would affect the accuracy of the detection kit. Results showed that the H_2_O_2_ concentration increased and the O_2_ content rapidly decreased to a low level with the increase of PTFL concentrations (Figure 1f). 200 µg mL^−1^ PTFL NPs could generate ≈101 µm H_2_O_2_ in 15 min. It is worth noting that when the concentration of PTFL NPs was higher than 120 µg mL^−1^, the increase rate of H_2_O_2_ slowed down. This could be explained by the low level of O_2_ that limited the rate of enzyme‐catalyzed reactions. When the PTFL NPs concentration was fixed at 200 µg mL^−1^ and NaL concentration was adjusted from 0 to 20 mm, the production of H_2_O_2_ showed a linear trend until the concentration of NaL exceeded 10 mm (Figure S6, Supporting Information). Figure S7, Supporting Information shows the O_2_ release curve of PTFL NPs in a normoxia environment for 24 h. A negligible decrease of O_2_ content in PTFL NPs was observed, demonstrating that PTFL NPs could contain O_2_ for a relatively long time in normoxia environment. As shown in Scheme 1, the natural O_2_‐carrying capacity of PFOB can promote the aerobic LOX enzymatic reaction. In the presence of O_2_‐carrying PTFL NPs and NaL (PTFL NPs +NaL), O_2_ content increased to 4.4 mg L^−1^ in the first 80 s and rapidly declined to only 0.9 mg L^−1^ due to the O_2_‐depleted LOX enzymatic reaction (Figure 1g). In the presence of only O_2_‐carrying PTFL NPs (PTFL NPs ‐NaL), O_2_ content increased to 4.9 mg L^−1^ in the first 80 s and then kept this high‐level O_2_ content due to no LOX enzymatic reaction (Figure 1g). For N_2_ gas‐treated PTFL NPs with NaL, O_2_ content was kept at a low level due to the lack of O_2_ supply (Figure 1g). Correspondingly, the final stable H_2_O_2_ production for the above three conditions after 380 s were also measured. As shown in Figure S8, Supporting Information, O_2_‐carrying PTFL NPs with NaL (PTFL NPs +NaL) showed a high H_2_O_2_ level due to the generation of H_2_O_2_ during LOX enzymatic reaction. N_2_ gas‐treated PTFL NPs with NaL and only O_2_‐carrying PTFL NPs (PTFL NPs ‐NaL) showed low H_2_O_2_ levels with no LOX enzymatic reaction due to the lack of O_2_ or lactate, respectively. The results indicate that the LOX enzymatic reaction is aerobic, and PTFL NPs can provide O_2_ to promote the LOX enzymatic reaction.

The stability of PTFL NPs in the normal environment (‐ATP) and the release of Fe ions from PTFL NPs caused by ATP (+ATP) were studied next. As shown in Figure 1e, ATP with strong chelation ability could degrade TA–Fe(III) shell to release Fe(III) ions, which were further reduced to Fe(II) ions by TA. It was reported that the ability of Fe(III) ions to catalyze H_2_O_2_ to produce ^•^OH was far inferior to that of Fe(II) ions. Moreover, during the generation of ^•^OH, Fe(II) ions would be re‐oxidized back to Fe(III) ions.^[^ ^32^, ^42^, ^43^ ^]^ Therefore, the release of Fe(III) ions by ATP and the continuous reduction of Fe(III) ions to Fe(II) ions by TA were both essential for the subsequent production of ^•^OH radicals. 1,10‐phenanthroline, which could coordinate with Fe(II) ions to form Fe(II)‐phenanthroline complexes with an absorption peak at 511 nm, was used to quantify the amount of Fe(II) ions (Figure S9, Supporting Information). Also, reductant hydroxylamine was used to convert Fe(III) ions to Fe(II) ions, so both Fe(III) and Fe(II) content could be quantified. As shown in Figure 1h, no Fe ions were released in the normal environment (‐ATP, black curve) and Fe ions were only released in the environment with ATP (+ATP, red curve). Moreover, most Fe ions (>85%) were released from PTFL NPs in the ATP environment within 1 h and 65% of Fe ions were reduced to Fe(II) ions at 6 h (green curve). The results demonstrated the stability of PTFL NPs in the normal physiological environment and the release of Fe ions in response to ATP. Importantly, elevated ATP expression is a common phenomenon that occurs only within the tumor cells. For in vivo experiments, PTFL NPs will not release TA–Fe(III) and LOX before accumulating in the tumor sites due to the stability in the normal physiological environment. When PTFL NPs accumulate at the tumor site and enter tumor cells, Fe(III) ions will be released in the elevated ATP environment, leading to tumor‐specific treatment.

Afterward, the generation of ^•^OH radicals catalyzed by Fe(II) ions was evaluated by ^•^OH caused methylene blue (MB) degradation. MB is a common oxidant indicator.^[^ ^38^ ^]^ When treated with ^•^OH radicals, the blue MB will fade with a significant decrease at the characteristic absorption peak at 665 nm. The declining absorption spectra of MB in Figure 1i showed that the ^•^OH radicals were continuously generated once PTFL NPs (200 µg mL^−1^) were mixed with NaL (10 mm) in the presence of ATP (1 mg mL^−1^). Importantly, no obvious fading of MB was observed in the absence of ATP, NaL, or neither (Figure 1j), demonstrating that both ATP and NaL were essential for PTFL NPs to generate ^•^OH radicals. In addition, ATP was significantly depleted when PTFL NPs were added into the solution containing ATP and NaL compared with the ATP and NaL solution group with no addition of PTFL NPs (Figure S10, Supporting Information). Hence, the whole process of depletion of lactate and producing ^•^OH can be summarized as follows: (1) PTFL NPs catalyze the lactate depletion and H_2_O_2_ generation in the supplying of O_2_ by PFOB; (2) Fe(III) ions are released by ATP from TA‐Fe(III) complexes and reduced to Fe(II) ions by TA; (3) The reduced Fe(II) ions catalyze H_2_O_2_ to generate ^•^OH radicals and deplete ATP, and are re‐oxidized to Fe(III) ions, which can be reduced again by TA for further ^•^OH generation.

### 2.3 In Vitro Synergistic Therapy

**Figure 2:**
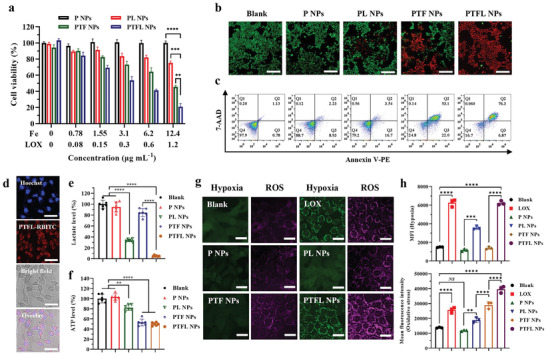
a) Cell viability of 4T1 cells after treatments with various concentrations of P NPs, PL NPs, PTF NPs, and PTFL NPs for 48 h (*n* = 3, mean ± SD). b) Confocal fluorescence images of calcein‐AM/PI stained 4T1 cells after various treatments (PTF NP, PTFL NPs: 72 µg mL^−1^). Scale bars: 200 µm. c) Apoptosis and necrosis analysis of 4T1 cells after various treatments in (b) from flow cytometry assay. d) Confocal fluorescence images of PTFL NPs endocytosis after 4 h incubation with 4T1 cells. PTFL NPs were tagged with RBITC (red). Scale bars: 50 µm. e,f) Lactate level in culture medium and intracellular ATP level of 4T1 cells after various treatments in (b) for 48 h (*n* = 6, mean ± SD). g) Hypoxia and ROS staining of 4T1 cells after various treatments of LOX, P NPs, PL NPs, PTF NPs, and PTFL NPs using CellROX reagent and Image‐iT Hypoxia reagent, respectively. Scale bars: 25 µm. h) Mean fluorescent intensity of both oxidative stress and hypoxia in 4T1 cells after various treatments in (g) from flow cytometry assay (*n* = 3, mean ± SD). Statistical significance was analyzed by one‐way ANOVA, *P < 0.05,**P < 0.01 and ****P < 0.0001, *NS*: nonsignificant difference.

After confirming the capacity of PTFL NPs to catalyze lactate decomposition and induce ^•^OH radicals in response to ATP, the in vitro therapeutic effect of the nanosystem was studied using CCK‐8 assay, live/dead staining assay, and apoptosis/necrosis flow cytometry assay. 4T1 cells were first divided into 6 groups and treated with (1) Blank, (2) P NPs, (3) PL NPs, (4) PTF NPs, and (5) PTFL NPs, respectively. Herein blank means no treatment. As shown in **Figure** **2**a, both PL NPs and PTF NPs decreased the cell viability modestly due to the lactate depletion and ATP depletion, respectively. The lowest cell viability in Group 5 manifested that the synergy of lactate depletion, ATP depletion, and CDT by employing PTFL NPs had the most anti‐cancer therapeutic effect (Fe content: 12.4 µg mL^−1^, LOX: 1.2 µg mL^−1^). Calcein‐AM/PI staining was then used to differentiate live and dead cells with green and red fluorescence, respectively. The confocal laser scanning microscopy (CLSM) results showed that 4T1 cells treated with PTFL NPs had the greatest degree of death (Figure 2b). Similar results of cell viability testing and live/dead fluorescence staining were also observed when PTFL NPs were applied for the treatment of CT26 murine colorectal carcinoma cell line (Figure S11, Supporting Information), indicating that PTFL NPs‐based therapy was applicable to different tumor types. In a contrast, MCF‐10A, a normal breast cell line, maintained high cell viability when incubated with various concentrations of PTFL NPs for 48 h, indicating a low cytotoxicity to normal cells (Figure S12, Supporting Information). Besides, flow cytometry assays were performed to evaluate the apoptosis/necrosis level of treated cells. As shown in Figure 2c, the cells treated with PTFL NPs had the highest degree of apoptosis. The total apoptosis ratio from Q2 and Q3 was 83.17%, while in P NPs, PL NPs, and PTF NPs‐treated groups, the total apoptosis ratios were 11.16%, 20.24%, and 75.1%, respectively. To determine the mechanism of PTFL NPs in causing apoptosis in 4T1 cells, western blotting of cleaved caspase‐3 protein was also performed. As shown in Figure S13, Supporting Information, PTFL NPs‐treated cells showed the highest level of cleaved caspase‐3 expression, indicating the apoptosis pathway by activation of caspases.

### 2.4 In Vitro Cellular Uptake, Lactate/ATP Depletion, and ROS/Hypoxia Generation

As mentioned, the synergistic therapeutic effects are based on LOX‐induced lactate depletion and H_2_O_2_ generation, as well as TA–Fe(III) complexes‐induced ATP depletion and ^•^OH radicals generation. Hence, in vitro lactate and ATP levels were measured to verify both depletions, while intracellular levels of ROS and hypoxia were investigated to confirm the ^•^OH generation and O_2_ supply/consumption. In vitro cellular uptake was first evaluated by incubating PTFL NPs with 4T1 cells. As shown in Figure 2d, rhodamine B isothiocyanate (RBITC) tagged PTFL (PTFL–RBITC) NPs underwent endocytosis by 4T1 cells after 4 h incubation. It was clearly observed that PTFL–RBITC entered the cells in the cytoplasm. Subsequently, in vitro lactate and ATP levels were investigated. As shown in Figure S14, Supporting Information, in vitro lactate concentration in cell culture medium kept increasing to ≈16.9 mm after 72 h incubation time. Then 4T1 cells were treated with P NPs, PL NPs, PTF NPs, and PTFL NPs, respectively. The relative lactate level showed no obvious change for the P NPs‐treatment group, while the lactate level in the PTF NPs treatment group decreased to ≈84.5% of that in the non‐treatment group. The lactate levels of PL NPs and PTFL treatment groups significantly decreased to 33.8% and 5.1%, respectively. The difference in the effect between PL NPs‐treatment group and PTFL NPs‐treatment group was mainly due to the different loading capacities of LOX (Figure 2e). Moreover, along with the depletion of lactate, intracellular ATP was also exhausted with the release of Fe(III) ions. As shown in Figure 2f and Figure S15, Supporting Information, the intracellular ATP levels in PTF NPs treatment group and PTFL NPs treatment group were markedly decreased to 52.9% and 49.8% of that without treatment respectively, demonstrating the successful depletion of intracellular ATP. Conversely, the groups without TA–Fe(III) component (P NPs, PL NPs) showed no significant ATP suppression. Similar ATP/lactate depletion results were also observed when PTFL NPs were applied for the treatment of CT26 cell lines (Figure S16, Supporting Information), demonstrating that PTFL NPs could achieve ATP/lactate depletion for the treatment of different tumor types.

**Figure 3:**
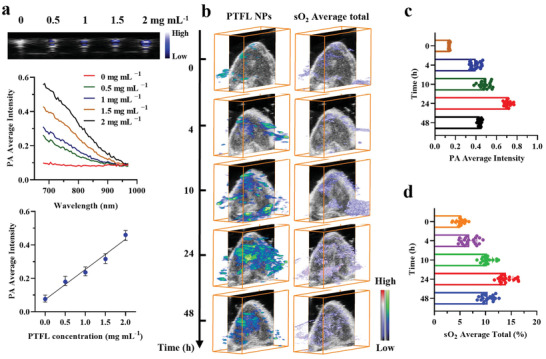
a) In vitro PA images and quantified intensities of PTFL NPs with various concentrations (*n* = 3, mean ± SD). b) In vivo 3D PA and sO_2_ tumor images at different time intervals after i.v. injection of PTFL NPs. c,d) The corresponding quantified intensities of PA and sO_2_ average total of tumor areas in (b) (*n* = 60, mean ± SD).

The LOX catalysis exhausted the in situ O_2_ content to generate local hypoxia, and the produced H_2_O_2_ was then catalyzed to ^•^OH radicals by released iron ions, a kind of ROS. The conversion from H_2_O_2_ to ^•^OH radicals further increase the total ROS level. Hence, intracellular hypoxia and ROS were both stained by fluorescent probes to show the change during NP treatments. Prior to the staining, the culture medium of 4T1 cells was covered with a layer of liquid paraffin to prevent the O_2_ exchange. As shown in Figure 2g, green color and red color represented the local hypoxia and ROS, respectively. As expected, P NPs could release the local hypoxia due to the O_2_‐sufficient PFOB cores. PTF NPs without LOX loading could only generate a low degree of ROS due to the insufficient intracellular H_2_O_2_. For PL NPs with LOX loading but no TA–Fe(III) coating, a certain extent of local hypoxia and ROS was generated during the LOX catalysis. Moreover, when NPs were decorated with both LOX and TA–Fe(III) coating, green and red fluorescence was enhanced obviously, which proved that PTFL NPs could produce a large amount of ROS and local hypoxia. As shown in **Figure** **3**h, the quantitative analysis from flow cytometry assay also showed the same trend that the mean fluorescence intensity (MFI) of oxidation stress of the cells treated with PTFL NPs was 3.7 times higher than the MFI of the control group, while the MFI of the hypoxia indicator was 4.2 times higher than that of the control group, respectively.

### 2.5 In Vitro and In Vivo PA Imaging Performance

The intrinsic high photothermal‐conversion capability of TA–Fe(III) complexes endowed PTFL NPs with enhanced PA contrast,^[^ ^44^ ^]^ therefore, PA imaging of PTFL NPs was further carried out both in vitro and in vivo. First, in vitro demonstration was performed using a tissue‐mimic phantom (Figure S17, Supporting Information). As shown in Figure 3a, PA amplitude increased with the concentration of PTFL NPs. The results of broad‐spectrum PA scanning showed that the PA amplitude of PTFL NPs in the range of 680 to 970 nm gradually weakened with the increase of wavelength, which was consistent with the results of Vis–NIR extinction. Herein, the PA signal excited by 700 nm laser was selected for in vivo PA imaging. Quantitative analysis revealed that the PA amplitudes of PTFL NPs were linearly correlated with the concentrations.

Afterward, in vivo tumoral PA imaging of PTFL NPs and the subsequent changes in saturated oxygen (sO_2_) were carried out via the intravenous (i.v.) administration to 4T1‐bearing nude mice. To better reveal the accumulation of PTFL NPs and the corresponding sO_2_ level throughout the tumor area, we performed PA tomographic scanning of the tumor and subsequent 3D imaging reconstruction. Briefly, each longitudinal section of the tumor was first scanned in turn, then the PA signals and sO_2_ levels of the tumor in each section were analyzed. Finally, 3D reconstruction was performed on PA images of all sections to enhance the visualization and accuracy of signals throughout the tumor. As shown in Figure 3b, after i.v. administration, PTFL NPs gradually accumulated in the tumor and reached a peak after 24 h, and then weakened a little after 48 h. The corresponding sO_2_ level displayed the same trend. The results revealed that PTFL NPs could well accumulate and retain in tumors. It is worth noting that after 24 h of administration, the signal of PTFL NPs evenly distributed in the interior and edge of the tumor, indicating a high permeability of PTFL NPs to the tumor as well. This might be explained that PTFL NPs were in a soft “nanodroplet” state and prone to deformation to increase penetration, which was different from solid NPs.^[^ ^49^, ^50^ ^]^ The high permeability was conducive to covering the entire tumor with our nanoagents, thereby improving the overall therapeutic effect.

Generally, the O_2_ loading capacity of NPs follows Henry's law, that is, proportional to the exposed O_2_ partial pressure. The high O_2_ loading capacity of our PTFL NPs is due to the high O_2_ solubility of PFOB, which has a very high 50% v/v (i.e., 500 mL_O2_/L_PFOB_) O_2_ solubility under the normal atmospheric O_2_ partial pressure.^[^ ^51^ ^]^ O_2_‐loaded PFCs‐based NPs can keep O_2_ encapsulated in the normoxia physiological environment for a long time due to the partial O_2_ pressure equilibrium and then release O_2_ in response to the hypoxia tumor environment due to the drop of exposed O_2_ partial pressure.^[^ ^52^ ^]^ In our case, in vivo sO_2_ level is affected by PTFL NPs from two aspects: the O_2_ supply by O_2_ self‐sufficient PFOB nanodroplets and O_2_ depletion by LOX‐based catalysis. Hence, PTF NPs without LOX addition were also introduced for comparison. After the i.v. administration of PTF NPs, the sO_2_ level in the tumor showed a similar upward‐decreasing trend (Figure S18, Supporting Information). The difference was that the peak sO_2_ level in PTFL NPs‐treated tumor was only 13.9%, whereas the value was increased to 20.4% in PTF NPs‐treated tumor. Therefore, it could be concluded that the self‐carrying O_2_ capacity of PFOB could increase the baseline of the tumor sO_2_ level.

### 2.6 In Vivo Fluorescent (FL) Imaging and Biodistribution

**Figure 4:**
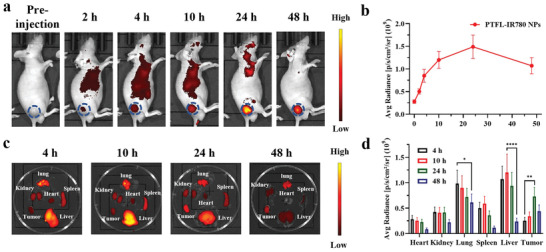
a) In vivo fluorescent images of 4T1 subcutaneous tumor‐bearing mice at various time intervals after i.v. injection of PTFL‐IR780 NPs and b) the corresponding fluorescent intensities of PTFL‐IR780 NPs at tumor sites (*n* = 3, mean ± SD). c) Ex vivo fluorescent images of major organs and tumors from 4T1 subcutaneous tumor‐bearing mice sacrificed at various time intervals after i.v. injection of PTFL‐IR780 NPs and d) the corresponding fluorescent intensities (*n* = 3, mean ± SD). Statistical significance was analyzed by one‐way ANOVA, *P < 0.05,**P < 0.01 and ****P < 0.0001, *NS*: nonsignificant difference.

IVIS spectrum imaging system was utilized for the in vivo FL imaging of PTFL NPs to study tumor accumulation and biodistribution. Prior to in vivo imaging, PTFL NPs were tagged with IR‐780 fluorescent dye (PTFL–IR780 NPs) to endow the nanoagent with FL imaging contrast. Visible‐near‐Infrared (Vis–NIR) extinction of PTFL–IR780 NPs is shown in Figure S19, Supporting Information, demonstrating the successful labeling of IR780. In addition, after 7 days of dispersion in PBS, the change in Vis–NIR extinction was found to be negligible, indicating that the labeling of IR780 was stable. The average NIR fluorescence signals of tumor sites from the IVIS images were quantified to evaluate the accumulation of PTFL–IR780 in the tumor sites. The average NIR fluorescence signals of tumor sites before the administration of PTFL‐IR780 were used as the baseline for comparison. As shown in **Figure** **4**a,b, obvious tumor fluorescence could be seen at 2 h post‐administration, indicating that PTFL–IR780 NPs started to accumulate at the tumor site. At 24 h of administration, the average NIR fluorescence signals of tumor sites continued to increase by 5.3 times compared to that before the administration, which demonstrated the successful accumulation of NPs in the tumor sites. The average NIR fluorescence signals of tumor sites at 48 h of administration only decayed by 28% compared to that at 24 h of administration, which demonstrated the good retention of NPs in the tumor sites. This result was consistent with that of in vivo PA imaging, which assisted in proving that PTFL NPs had both excellent tumor accumulation and long‐term retention capability. At different time intervals (4 h, 10 h, 24 h, and 48 h), 4T1‐bearing nude mice were sacrificed. Major organs and tumors were excised and performed ex vivo IVIS imaging. It could be observed that PTFL NPs also accumulated in livers and lungs except for tumors (Figure 4c,d). The amount of PTFL NPs in major organs dropped to a very low level within 48 h, probably due to the clearance of TA–Fe(III) complexes and the exhalation of PFOB nanodroplets.^[^ ^53^, ^54^ ^]^

### 2.7 In Vivo Synergistic Therapy

**Figure 5:**
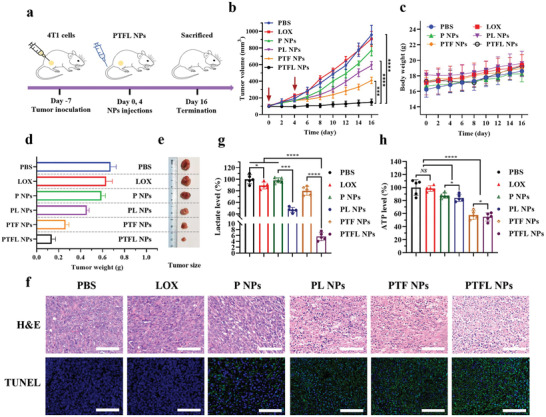
a) Schematic illustration of in vivo therapy for 4T1 subcutaneous tumor‐bearing mice. b) Tumor volumes, c) body weights, d) tumor weights, and e) representative tumor images of 4T1 tumor‐bearing nude mice after various treatments for 16 days (*n* = 3, mean ± SD). Arrows indicate the time point of treatments (Days 0 and 4). f) H&E staining and TUNEL immunofluorescence staining (Green) images after various treatments. Scale bars: 100 µm. g,h) Lactate and ATP levels in 4T1 tumors with various treatments 48 h after administrations (*n* = 5, mean ± SD). Statistical significance was analyzed by one‐way ANOVA, *P < 0.05,**P < 0.01 and ****P < 0.0001, *NS*: nonsignificant difference.

PTFL NPs have proven their excellent anti‐tumor capability in vitro, as well as their satisfactory tumor‐targeting capability in vivo. Next, the in vivo synergistic therapeutic effect was evaluated on 4T1 subcutaneous tumor‐bearing mice. Mice were randomly divided into 6 groups and treated with PBS, LOX, P NPs, PL NPs, PTF NPs, and PTFL NPs at days 0 and 4, respectively, and sacrificed at day 16 for further analysis (**Figure** **5**a). As shown in Figure 5b, tumors in mice treated with PTFL NPs almost disappeared, whereas in mice treated with P NPs, PL NPs, and PTF NPs, tumors were only partially destroyed. Mice treated with free LOX showed no obvious tumor suppression compared to PBS treated mice because of the fast clearance and poor tumoral accumulation. The tumor growth inhibition rate in each treatment was 5.1% (free LOX), 19.5% (P NPs), 38.5% (PL NPs), 64.4% (PTF NPs), and 84.5% (PTFL NPs), respectively. No abrupt changes in body weights were found for all the groups (Figure 5c). Tumor weights and digital graphs of representative tumors after excision supported the same trend (Figure 5c,d). Hence, it can conclude that PTFL NPs suppress tumor growth to the utmost extent based on the cascade lactate/ATP depletion and synergistic CDT. Furthermore, hematoxylin and eosin (H&E) staining and terminal deoxynucleotidyl transferase dUTP nick end labeling (TUNEL) immunostaining were performed as auxiliary proof from the pathological analysis (Figure 5f). It could be observed that the tumor treated with PTFL NPs underwent the most cell disruption and shrinkage with the largest nucleus absence in H&E staining, and had the densest fluorescence in TUNEL assay. The results revealed that PTFL NPs produced the most cell apoptosis. Whereas in tumors treated with PTF NPs, PL NPs, or P NPs, the degrees of cell apoptosis decreased in various degrees. Tumors treated with PBS or free LOX still exhibited complete cell morphology with the large nucleus and no obvious TUNEL fluorescence, indicating that the tumors were still in an uninhibited and malignant state. Besides, the biosafety of PTFL NPs was also evaluated. As shown in Figure S20, Supporting Information, no obvious histological damage was found in major organs of mice in each group, illustrating the negligible systemic toxicity as expected. All the above results demonstrate that PTFL NPs could maximally inhibit the tumor growth with negligible systemic toxicity by the cascade metabolic‐CDT based on lactate/ATP depletion and ^•^OH radicals generation.

### 2.8 In Vivo TME Regulation, Anti‐Angiogenesis, and Anti‐Metastasis Performance

Afterward, the in vivo mechanism of LOX‐induced tumor inhibition was evaluated. Tumoral lactate/ATP level, hypoxia‐related biomarker expression (HIF1‐*α*), angiogenesis biomarkers expression (CD31 and VEGF), as well as lung metastasis were investigated correspondingly. First, tumoral lactate/ATP concentrations were measured 48 h post‐treatment with PBS, free LOX, P NPs, PL NPs, PTF NPs, and PTFL NPs. For tumor lactate depletion, the results revealed that the tumoral lactate was almost exhausted with the depletion rate >90% in PTFL NPs‐treated group, demonstrating the excellent catalytic activity of LOX‐embedded PTFL NPs (Figure 5g). PL NPs showed a partial lactate depletion rate of ≈53.5%. Free LOX could not accumulate in tumors and P NPs/PTF NPs had no lactate depletion effect, hence the decrease of lactate level was not obvious in these three groups. For tumor ATP depletion, the results revealed that 42.4% and 45.2% of tumoral ATP was suppressed in PTF NPs and PTFL NPs‐treated groups respectively (Figure 5h). P NPs and PL NPs without TA‐Fe(III) components only showed a slight ATP decrease because of suppression of tumor growth, while free LOX with poor tumoral accumulation could not suppress ATP level. Next, the HIF1‐*α* (green fluorescence) as a tumor hypoxia indicator was immunostained. As shown in Figure S21, Supporting Information, the PBS/free LOX‐treated tumors showed obvious hypoxia with a high expression of HIF1‐*α*, whereas in P NPs/PTF NPs‐treated tumors, almost no green fluorescence was observed, indicating the relief of hypoxia due to O_2_ supplying by PFOB component. The PL NPs/PTFL NPs‐treated tumor showed restrained HIF1‐*α* expression which was attributed to the combined effect of O_2_ delivery by PFOB and O_2_ depletion by LOX.

**Figure 6:**
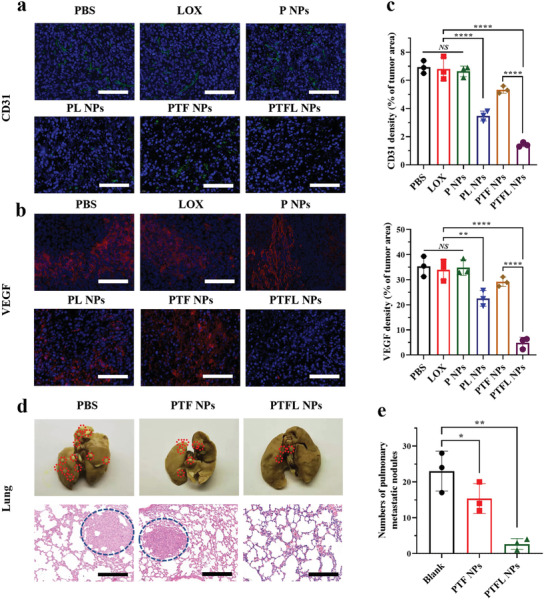
a) CD31 and b) VEGF immunostaining images and c) the corresponding quantification of CD31 and VEGF densities (*n* = 3, mean ± SD). Scale bars: 100 µm. d) Representative images of tumor nodules in the lungs with various treatments and e) the corresponding number of lung metastasis nodules (*n* = 3, mean ± SD). Scale bars: 200 µm. Statistical significance was analyzed by one‐way ANOVA, *P < 0.05,**P < 0.01 and ****P < 0.0001, *NS*: nonsignificant difference.

Sequentially, two angiogenesis biomarkers (CD31 and VEGF) were immunostained to investigate the anti‐angiogenesis efficacy. As shown in the fluorescent images from **Figure** **6**a,b and the semi‐quantitative analysis from Figure 6c, a large amount of green and red positive spots were observed in the groups treated with PBS, free LOX, and P NPs, indicating a high level of CD31 and VEGF expression. PTF NPs treatment slightly reduced both expression levels due to effective tumor suppression. PL NPs treatment distinctly decreased both expressions and for PTFL NPs treatment, areas of green and red dots dropped significantly by 67% and 86%, respectively, indicating the anti‐angiogenesis effect of lactate depletion by the loaded LOX. Next, the anti‐metastasis effect of PTFL NPs was evaluated. 4T1 cells were planted under the armpit to facilitate tumor metastasis. Mice were treated with PBS, PTF NPs, or PTFL NPs. As shown in Figure 6d, 21 days after the first administration, obvious metastasis nodules could be observed on the digital and H&E staining images of lungs in PBS‐treated group. Compared with the PBS‐treated control group, the mice treated with PTF NPs exhibited less metastasis while for the mice treated with PTFL NPs, lung metastasis nodules were almost negligible. Quantitative analysis (Figure 6e) demonstrated that PTFL NPs could prominently reduce the metastasis nodules by 89%. Hence, this nanosystem was proven to have effective anti‐angiogenesis and anti‐metastasis effects.

## 3 Conclusion

In summary, an O_2_ self‐supplying theranostic nanoagent (PTFL NPs) with cascade catalytic reaction was developed for synergistic metabolic‐CDT by dual‐depletion of lactate and ATP together with the conversion of H_2_O_2_ to ^•^OH radicals. Specifically, PTFL NPs could efficiently deplete tumor lactate with the assistance of O_2_‐sufficient PFOB core, and generate H_2_O_2_ to supplement the insufficient endogenous H_2_O_2_. Synergistically, PTFL NPs responded to overexpressed intracellular ATP in tumor cells to deplete ATP and converted H_2_O_2_ to cytotoxic ^•^OH radicals. In vivo studies revealed that under the guidance of PA imaging, the PTFL NPs had excellent tumor accumulation and retention for suppression of tumor growth, as well as showed the anti‐angiogenesis and anti‐metastasis effects. This unique lactate/ATP dual‐depletion cascade catalytic nanosystem with the generation of cytotoxic ^•^OH radicals might provide new insight for synergistic metabolic‐chemodynamic tumor therapy.

## 4 Experimental Section

### Materials

PFOB, Pluronic F‐127, Iron (III) chloride hexahydrate (FeCl_3_ · 6H_2_O), LOX from *Aerococcus viridans*, 2′,7′‐Dichlorofluorescin diacetate (DCFH‐DA), IR‐780 iodide, and rhodamine B isothiocyanate (RBITC) were purchased from Sigma Aldrich (USA). Methylene blue (MB) was purchased from TCI (Japan). Hydroxylamine hydrochloride, sodium lactate (NaL), and tannic acid (TA) were purchased from J&K Scientific (China). ATP and 1,10‐phenanthroline were purchased from Aladdin Reagent (China). Hydrogen peroxide (H_2_O_2_) assay kit, cell counting KIT‐8 (CCK‐8), ATP assay kit, and lactate assay kit were purchased from Solarbio (China). Live/dead cell imaging kit, image‐iT hypoxia reagent, cellROX deep red reagent, Hoechst 33342, and all cell culture reagents were purchased from ThermoFisher Scientific (USA). PE Annexin V Apoptosis Detection Kit I was purchased from BD Biosciences (USA). All reagents and chemicals were purchased commercially and without further purification.

### Instrumentation and Characterization

An Ultrospec 2100 Pro spectrophotometer (Amersham Biosciences, UK) was utilized to measure the Vis–NIR extinction. A Zetasizer Nano ZS (Malvern Instruments, UK) was utilized to measure zeta potential and hydrodynamic size. A Field Emission Electron Microscope STEM (JEM‐2100F, JEOL Ltd., Japan) was utilized to observe the morphology of NPs. A Leica TCS SPE Confocal Microscope (Leica Microsystems, Germany) was utilized for in vitro fluorescence imaging. An IVIS Lumina Series III Pre‐clinical In Vivo Animal Imaging Systems (PerkinElmer, USA) was utilized for in vivo fluorescent imaging. A VEVO LAZR Imaging System (FUJIFILM VisualSonics, Canada) operating with an LZ250 transducer was utilized for PA imaging.

### Synthesis of P NPs, PL NPs, PTF NPs, and PTFL NPs

For P NPs, and PTF NPs synthesis, 100 µL PFOB was first added into 10 mL ice‐cold F‐127 solution (1% w/w). After 3 min emulsification by sonication (300 W, duty cycle 50%) in an ice‐water bath, P NPs were obtained. Then FeCl_3_ · 6H_2_O was mixed at a final concentration of 0.6 mg mL^−1^, and the formed PFOB–Fe(III) solution kept shaking at 1400 rpm overnight. Afterward, TA was quickly added under vigorous stirring with a final concentration of 1.2 mg mL^−1^. The solution instantly turned from faint yellow to blue and was further centrifuged three times at 7000 rpm for 3 min to obtain PTF NPs. For PL NPs synthesis, LOX was first mixed with P NPs without the addition of FeCl_3_ · 6H_2_O and TA. After shaking overnight and washing, PL NPs were obtained. For PTFL NPs synthesis, the recipe changed that LOX was first mixed along with FeCl_3_ · 6H_2_O. After the successful emulsification of PFOB nanodroplets, the mixture of LOX and FeCl_3_ · 6H_2_O were added at a final concentration of 0.8 and 0.6 mg mL^−1^ respectively. After shaking overnight, the PFOB–Fe(III)–LOX solution was quickly mixed with TA to get PTFL NPs. Subsequently, the obtained PTFL NPs solution was centrifuged at 7000 rpm for 3 min and washed three times with distilled water. To evaluate the amount of loaded active LOX, H_2_O_2_ produced by LOX catalysis was measured using an H_2_O_2_ assay kit based on a standard H_2_O_2_ curve. Finally, the purified PTFL NPs were stored at 4 °C. Before all subsequent experiments, the prepared PTFL NPs were put in an aseptic chamber and exposed to a normoxia environment for 2 h. Due to the high O_2_ solubility of PFOB, O_2_ molecules then entered the NPs until the O_2_ saturation and O_2_ partial pressure balance were reached.

### Catalytic Activity Measurements of PTFL NPs

The generation of H_2_O_2_ and depletion of O_2_ were detected to determine the catalytic activity of PTFL NPs. Various concentrations of PTFL NPs from 0 to 200 µg mL^−1^ were first mixed with NaL (10 mm) in PBS and incubated for 15 min. Then the concentration of H_2_O_2_ and O_2_ was measured by an H_2_O_2_ Assay kit and a portable dissolved O_2_ meter, respectively. The standard curve of H_2_O_2_ was determined according to the manufacturer's instruction. Also, the influence of NaL concentration (0, 2.5, 5, 10, 20 mm) on H_2_O_2_ generation by PTFL NPs was investigated. Last, the promotion of LOX catalytic reaction by O_2_ carried by PFOB core was also explored. An aqueous solution with or without NaL addition was pre‐treated with N_2_ to exhaust O_2_, and sealed with a layer of liquid paraffin to prevent air exchange. Then 200 µg mL^−1^ of PTFL NPs with or without N_2_ gas treatment were injected, and the change of O_2_ content was monitored throughout the process to reflect the PFOB‐assisted LOX catalysis.

### Iron Ions Release and ^•^OH Generation

Iron ions release in response to ATP was determined according to the previous study.^[^ ^40^ ^]^ Briefly, 0.2 mg mL^−1^ PTFL NPs were mixed with 1 mg mL^−1^ ATP under continuous shaking. At different time intervals, PTFL NPs solution was centrifuged and the supernatant was divided, then added with distilled water to half and hydroxylamine hydrochloride (1 mg mL^−1^) to the other half for 15 min. After that, the reacted aqueous solutions were mixed with 1,10‐phenanthroline (1 mg mL^−1^), and the absorptions at 510 nm were measured to calculate the release amount of total iron ions and converted Fe(II) ions based on a standard curve. To investigate the ^•^OH generation, MB degradation by ^•^OH was measured as an indicator according to the absorption at 665 nm. Briefly, 0.2 mg PTFL, 1 mg ATP, and 10 µg MB were mixed in 1 mL NaL (10 mm) and incubated at room temperature. At different time points (0, 10, 20, 30, 40, 50, 60 min), the mixture was centrifuged and absorption of the supernatant was measured. Besides, four different combination trials were designed to prove that the MB degradation was caused by the combined effect of PTFL, lactate, and ATP. The first group contained 0.2 mg mL^−1^ PTFL only, while the second group contained both PTFL (0.2 mg mL^−1^) and ATP (1 mg mL^−1^). The third group consisted of PTFL (0.2 mg mL^−1^) and NaL (10 mm), and the last group consisted of PTFL (0.2 mg mL^−1^), NaL (10 mm), and ATP (1 mg mL^−1^). The absorption of MB at 665 nm was measured at different time intervals, which indicated the level of ^•^OH generation in each group. Also, ATP levels were detected using an ATP assay kit.

### Cell Culture

Murine mammary carcinoma 4T1 cells, murine mammary carcinoma 4T1 cells, and human non‐tumorigenic epithelial cell line MCF‐10A cells were all purchased from the American Type Culture Collection (ATCC, Manassas, VA). Murine mammary carcinoma 4T1 cells were used for in vitro cytotoxicity assay (Figure 2a), fluorescence live/dead assay (Figure 2b), flow cytometry (Figure 2c), fluorescence imaging of PTFL NPs endocytosis (Figure 2d), measurement of lactate level in culture medium and intracellular ATP level after various treatments (Figure 2e,f), hypoxia and ROS fluorescence staining (Figure 2g), fluorescent intensity analysis of both oxidative stress and hypoxia after various treatments (Figure 2h), western blot analysis of the expression levels of cleaved caspase‐3 (Figure S13, Supporting Information), measurement of lactate accumulation at different time intervals (Figure S14, Supporting Information), and intracellular ATP level treated with various concentrations of PTF NPs (Figure S15, Supporting Information). Murine colorectal carcinoma CT26 cells were used for in vitro cytotoxicity assay (Figure S11a, Supporting Information), fluorescence live/dead assay (Figure S11b, Supporting Information), and measurement of lactate level in culture medium and intracellular ATP level after various treatments (Figure S16, Supporting Information). Human non‐tumorigenic epithelial cell line MCF‐10A cells were used for in vitro cytotoxicity assay (Figure S12, Supporting Information). Both 4T1 cells and CT26 cells were cultured in RPMI 1640 supplemented with 10% heat‐inactivated fetal bovine serum (FBS), penicillin (100 units mL^−1^), and streptomycin (100 mg mL^−1^) at 37 °C in a humidified atmosphere with 5% CO_2_. MCF‐10A cells were cultured in complete growth medium including DMEM/F‐12 medium supplemented with horse serum (5%), epidermal growth factor (20 ng mL^−1^), insulin (10 µg mL^−1^), hydrocortisone (500 ng mL^−1^), penicillin/streptomycin (1 unit mL^−1^), and cholera toxin (0.1 µg mg^−1^).

### Cell Viability Test

4T1 cells were seeded in 96‐well plates at a density of 5 × 10^3^ cells per well. After 24 h, all the cell culture medium was replaced by fresh ones containing different concentrations of P NPs, PL NPs, PTF NPs, or PTFL NPs. Herein, the same concentration of P NP, PL NPs, PTF NPs, and PTFL NPs refers to the same content of PFOB. The whole co‐culture time lasted for 48 h, then the CCK‐8 assay kit was used to detect the cell viability by a microplate reader (Thermo Scientific, Varioskan LUX, USA). Besides, live & dead fluorescent imaging of treated cells was realized by calcein‐AM and PI staining. Briefly, after 24 h culture of 4T1 cells, the culture medium was replaced with fresh ones containing different NPs. 10 h later, all groups were stained by calcein‐AM and PI and further observed under a CLSM. Experiments were repeated that 4T1 cells were replaced with CT26 cells and all the conditions were kept the same.

### Apoptosis & Necrosis Assay

Annexin V‐PE/7‐AAD apoptosis detection kit was used to investigate the cell apoptosis induced by P NPs, PL NPs, PTF NPs, and PTFL NPs. The co‐culture of 4T1 cells and NPs was kept for 12 h, then cells in each group were carefully harvested. The harvested cells were counted using a cell counting chamber, treated with Annexin V‐PE/7‐AAD apoptosis detection kit, and analyzed by a flow cytometer according to the manufacturer's instruction. Besides, western blot analysis was also conducted with the same treatments on 4T1 cells with GAPDH as control.

### Cell Endocytosis Test

The cellular uptake of PTFL NPs was investigated by incubating PTFL NPs with 4T1 cells. To ensure PTFL NPs could be observed under CLSM, PTFL NPs were first tagged with RBITC (*λ*_em_ = 580 nm). All the synthesis steps were the same except that 1 µL RBITC (20 mg mL^−1^) was added in PFOB emulsion together with FeCl_3_ · 6H_2_O. 4T1 in a total amount of 5 × 10^4^ were first seeded in a 35 mm confocal dish. After 24 h, the culture medium was replaced by a fresh one containing RBITC tagged PTFL (PTFL–RBITC) NPs (120 µg mL^−1^). The cells were incubated for another 4 h and washed with PBS three times, then 1 µg mL^−1^ Hoechst 33342 was added to label the nucleus for 15 min. Subsequently, CLSM images were taken to observe the endocytosis of PTFL NPs.

### Intracellular ROS and Hypoxia Detection

The generation of PTFL NPs induced oxidative stress and hypoxia was detected by Image‐iT Hypoxia Reagent (Invitrogen) and CellROX Deep Red Reagent (Invitrogen). First, 4T1 cells were seeded in 96‐well plates at a density of 5 × 10^3^ cells per well. After 24 h, the culture medium was replaced by a fresh one containing P NP, PL NPs, PTF NPs, or PTFL NPs (120 µg mL^−1^) with a layer of liquid paraffin coverage to prevent the O_2_ exchange, then co‐cultured for another 6 h. Subsequently, 4T1 cells in each group were washed with PBS three times and stained by hypoxia and oxidative stress probes. CLSM images were taken to qualitatively analyze the intracellular level of hypoxia and oxidative stress in each group. Quantitative analysis of intracellular ROS and hypoxia was obtained by flow cytometry according to the manufacturer's instructions. The cells without any treatments were set as blank.

### In Vitro Lactate Generation & Depletion

Lactate generation at different time points (12, 24, 48, 72 h) during 4T1 cells growth was evaluated by measurement of lactate level in cell supernatant using the lactate assay kit. For lactate depletion detection under various treatments, after incubation for 24 h, 4T1 cells were treated with P NP, PL NPs, PTF NPs, or PTFL NPs for another 24 h. Thereafter, the supernatants were collected to measure the lactate concentration. Experiments were repeated with CT26 cells and all the conditions were kept the same.

### In Vitro ATP Depletion

4T1 cells were seeded in 96‐well plates at a density of 5 × 10^3^ cells per well. After 24 h, the culture medium was replaced by a fresh one containing P NPs, PL NPs, PTF NPs, or PTFL NPs, and incubated for another 24 h. Thereafter, the 4T1 cells were collected, disrupted, and analyzed using an ATP assay kit for the measurement of intracellular ATP levels. Also, different concentrations of PTF NPs were used for 4T1 treatment, and the intracellular ATP levels were measured in the same manner.

### Animals and Tumor Model

BALB/c nude mice (4–6 weeks old, female or male) were purchased from Centralized Animal Facilities of The Hong Kong Polytechnic University. All animal protocols were conducted under the Guidelines for Care and Use of Laboratory Animals of Department of Health of the Government of the Hong Kong Special Administrative Region. An animal ethics approval was obtained from the Animal Ethics Committee of The Hong Kong Polytechnic University (Ref No.: 20‐21/124‐BME‐R‐GRF) with an animal license issued by the Department of Health of the Government of the Hong Kong Special Administrative Region (Ref No.: (20–62) in DH/HT&A/8/2/4 Pt.2). For in vivo anti‐tumor and biodistribution study, 4T1 cells were harvested and suspended in the serum‐free medium at a density of 2 × 10^7^ cells mL^−1^. Then 0.1 mL cell suspension was injected subcutaneously to the back of mice. For in vivo anti‐metastasis study, 4T1 cells were collected and injected into the right armpit of mice (2 × 10^7^ cells mL^−1^, 0.1 mL).

### In Vitro/In Vivo PA Imaging

In vitro demonstration of PA imaging was carried out in tissue‐mimic phantom using polyethylene (PE) tubes (0.5 mm inner diameter). Briefly, PTFL NPs with various concentrations from 0 to 2 mg mL^−1^ were injected into PE tubes, then all PE tubes were immersed in water for PA imaging. Both single wavelength scanning (700 nm) and spectra scanning (680–970 nm) were utilized to analyze the PA performance of PTFL NPs. For in vivo PA imaging study, after the tumor volume reached 100 mm^2^, 0.2 mL PTF NPs and PTFL NPs (1 mg mL^−1^) were intravenously (i.v.) injected into different 4T1 subcutaneous tumor‐bearing mice. At different time intervals (0, 4, 10, 24, 48 h), PA tomographic scanning was operated at 700 nm for the investigation of PTF/PTFL NPs accumulation, while oxygen saturation (sO_2_) scanning was operated at 750/850 nm for the analysis of tumor oxygenation level. Further quantitative data analysis and 3D reconstruction were performed on FUJIFILM VEVO software (FUJIFILM VisualSonics, Canada).

### In Vivo Fluorescent Imaging and Biodistribution

IR‐780 fluorescent dye was introduced to tag PTFL NPs (PTFL–IR780) for in vivo fluorescence imaging. IR‐780 was only used for in vivo fluorescence imaging experiments and was not used for in vivo therapy experiments. All the synthesis steps were the same as PTFL NPs synthesis except that 5 µL IR‐780 (10 mg mL^−1^ in DMSO) was added in PFOB emulsion together with FeCl_3_ · 6H_2_O. The obtained PTFL–IR780 NPs were centrifuged to remove the unloaded IR‐780 and re‐dispersed in PBS. The successful labeling of IR780 and the stability of the labeling method were characterized by measuring the Vis–NIR extinction of the PTFL–IR780 NPs before and after dispersing in PBS for 7 days. After the tumor reached an approximate size of 100 mm^3^, 4T1 subcutaneous tumor‐bearing mice were i.v. injected with 0.2 mL PTFL–IR780 NPs (1 mg mL^−1^), then imaged under an IVIS system with an excitation wavelength of 760 nm and an emission wavelength of 845 nm at different time intervals (0, 2, 4, 10, 24, 48 h). For the biodistribution study, the PTFL‐IR780 treated mice were sacrificed at 4, 10, 24, 48 h post‐administration. The major organs (lungs, kidneys, hearts, spleens, and livers) and tumor tissues were excised and imaged ex vivo with the same excitation (760 nm) and emission (845 nm). The PerkinElmer Living Image Analysis Software was used to further conduct the region of interest analysis.

### In Vivo Therapeutic Efficacy

The 4T1 subcutaneous tumor‐bearing mice were divided into 6 groups for different treatments as follows: 1) PBS, 2) LOX, 3) P NPs, 4) PL NPs, 5) PTF NPs, 6) PTFL NPs at an equivalent dose of 2.12 mg kg^−1^ of Fe and 0.21 mg kg^−1^ LOX. It should be noted that group (2) and (6) has the same amount of LOX, and for group (3), (4), (5) and (6), the amount of PFOB was the same. All the treatments were based on i.v. administration. Besides, the other treatments were performed on day 4 after the first treatments to ensure therapeutic efficacy. Tumor size and body weights were measured every 2 days. Tumor volumes were calculated as length × width^2^ /2. After 16 days post‐treatment, all the mice were sacrificed, and the tumors were excised and weighted.

### TME Investigation

After the 4T1 subcutaneous tumor‐bearing mice were divided into 6 groups (3 mice each group) after the tumors reached an approximate size of 100 mm^3^. Then each group was treated with 200 µL PBS, LOX, P NPs, PL NPs, PTF NPs, and PTFL NPs. At 48 h post‐administration, all the mice were sacrificed, and tumors were collected. Subsequently, the tumors were first lysed using a Precellys Evolution tissue homogenizer (Bertin Instruments, France), then the supernatants were collected, and the concentration of lactate and ATP was measured using the lactate assay kit and ATP assay kit according to the manufacturer's instructions.

### Anti‐Metastasis Evaluation

The 4T1 axillary tumor‐bearing mice were introduced to evaluate the anti‐metastasis effect. Briefly, mice were randomly assigned to 3 groups (*n* = 3) and treated with PBS, PTF NPs (0.2 mg), and PTFL NPs (0.2 mg) respectively at days 0, 4, and 9. 21 days after the first treatment. Mice were sacrificed, and lungs were collected and fixed by Bouin's solution. Subsequently, representative digital photographs were taken to observe the lungs and the number of pulmonary metastatic nodules were recorded. Eventually, H&E staining was used to further observe the metastasis in the lungs.

### Histology and Immunofluorescence Staining

On day 16 post‐treatment, the mice in different groups were sacrificed and the major organs (lungs, kidneys, hearts, spleens, and livers) and tumor tissues were collected and fixed in 4% paraformaldehyde. First, the fixed tumor tissues were sliced and stained with H&E, terminal deoxynucleotidyl transferase dUTP nick end labeling (TUNEL) according to the instructions. Besides, immunofluorescence staining against hypoxia‐inducible factor 1‐alpha (HIF1‐*α*), vascular endothelial growth factor A (VEGF‐A), cluster of differentiation 31 (CD31) biomarkers was performed to investigate the generation of tumor hypoxia, the anti‐angiogenesis, and the anti‐metastasis effects. The nuclei were stained with DAPI. All the slices were then imaged by optical microscopy or CLSM. Eventually, the major organs (lungs, kidneys, hearts, spleens, and livers) were sectioned and stained with H&E, followed by the observation under optical microscopy to confirm the biocompatibility of treated materials. Blinded histological analysis of the stained tissue slices was assessed by a trained surgeon at The First Affiliated Hospital, College of Medicine, Zhejiang University.

### Statistical Analysis

Student's *t*‐test was utilized for single comparisons and one‐way analysis of variance (ANOVA) was utilized for multiple comparisons. All statistical analyses were performed using GraphPad Prism 8 software. Significant differences were defined as *P < 0.05, **P < 0.01, ***P < 0.001, and ****P < 0.0001 while non‐significant (NS) differences were defined as p > 0.05. All data were expressed as mean ± standard deviation (SD).

## Conflict of Interest

The authors declare no conflict of interest.

## Supporting information

Supporting Information

## Data Availability

Research data are not shared.
