# Supplementary material for: Dual‐Depletion of Intratumoral Lactate and ATP with Radicals Generation for Cascade Metabolic‐Chemodynamic Therapy
Source: Adv Sci (Weinh). 2021 Oct 29;8(24):2102595. doi: 10.1002/advs.202102595 (PMC8693033; doi:10.1002/advs.202102595)
Supplement: Supplementary file 1 — Supporting Information [file ADVS-8-2102595-s001.pdf]

WILEY-VCH

© 2021 Wiley-VCH GmbH

ADVANCED SCIENCE
Open Access

Supporting Information

for *Adv. Sci.,* DOI: 10.1002/advs.202102595

Dual-Depletion of Intratumoral Lactate and ATP with Radicals Generation for Cascade Metabolic-Chemodynamic Therapy

*Feng Tian, Shiyao Wang, Keda Shi, Xingjian Zhong, Yutian Gu, Yadi Fan, Ruolin Zhang, Yu Zhang and Mo Yang**

Supporting Information

Dual-Depletion of Intratumoral Lactate and ATP with Radicals Generation for Cascade Metabolic-Chemodynamic Therapy

*Feng Tian, Shiyao Wang, Keda Shi, Xingjian Zhong, Yutian Gu, Yadi Fan, Ruolin Zhang, Yu Zhang and Mo Yang**

Table 1. Lactate and glucose concentration in the tumor, blood, or normal tissue.

	Tumor	Blood or normal tissue	Ref
Lactate concentration	10-30 mM	1.5-3 mM	[24-26]
Glucose concentration	0.1–0.4 mM	6 mM	[23]

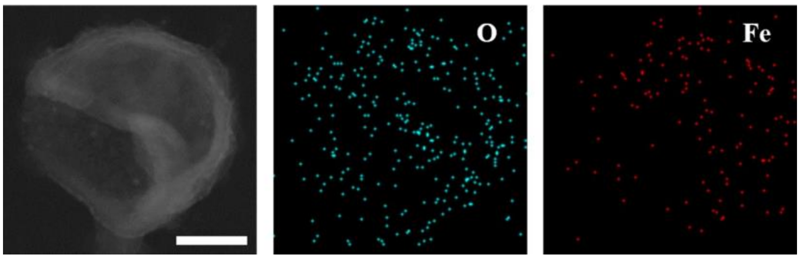

Figure S1. EDS elemental mapping of PTFL NP. Scale bar: 50 nm

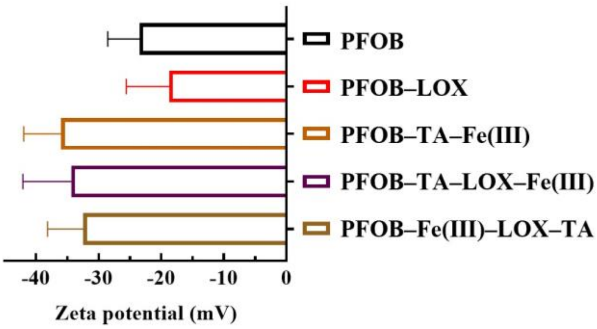

Figure S2. Zeta potential of PFOB, PFOB–LOX, PFOB–TA–LOX, PFOB–Fe(III)–LOX, PFOB–TA–Fe(III), PFOB–TA–LOX–Fe(III), PFOB–Fe(III)–LOX–TA NPs (n = 3, mean ± SD).

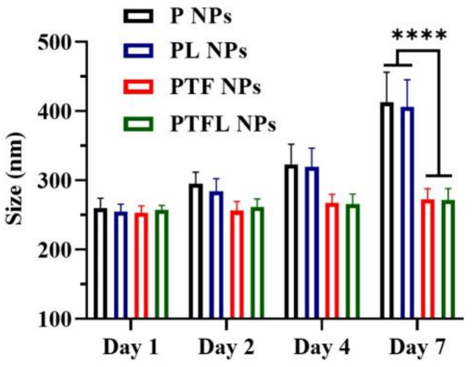

Figure S3. Size stability tests of P NPs, PL NPs, PTF NPs, and PTFL NPs (n = 3, mean ± SD). Statistical significance was analyzed by one-way ANOVA (****P < 0.0001).

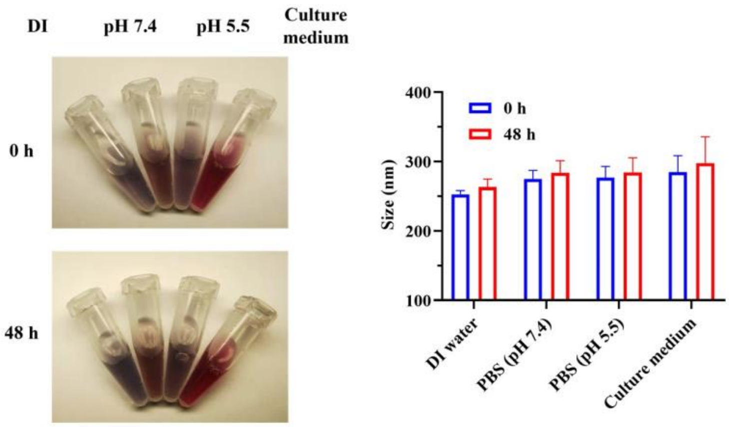

Figure S4. Digital pictures and DLS measurement of PTFL NPs solution dispersed in DI water, PBS buffer (pH = 7.4), PBS buffer (pH = 5.5), and cell culture medium before and after 48 h (n = 3, mean ± SD).

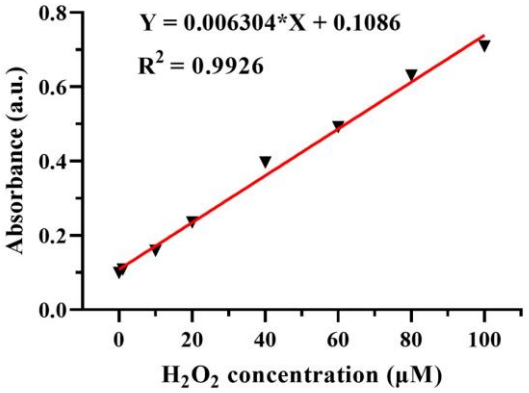

Figure S5. The standard curve of the H_2O_2 assay kit (n = 3, mean ± SD).

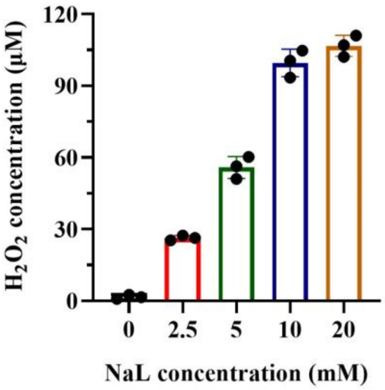

Figure S6. NaL concentration-dependent H_2O_2 generation by PTFL NPs (n = 3, mean ± SD).

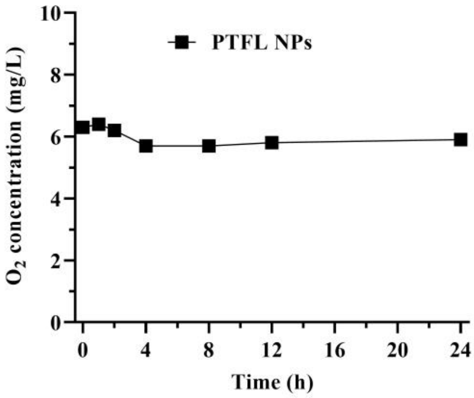

Figure S7. O_2 retention of PTFL NPs under normoxia environment.

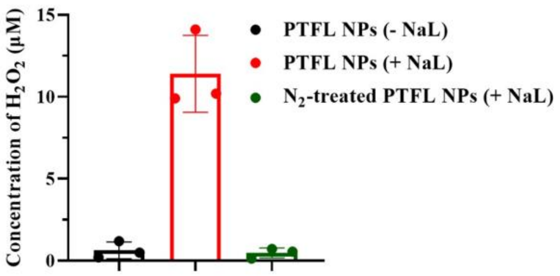

Figure S8. H_2O_2 generation after adding PTFL NPs to N_2-treated solution (with/without 10 mM NaL) (n = 3, mean ± SD).

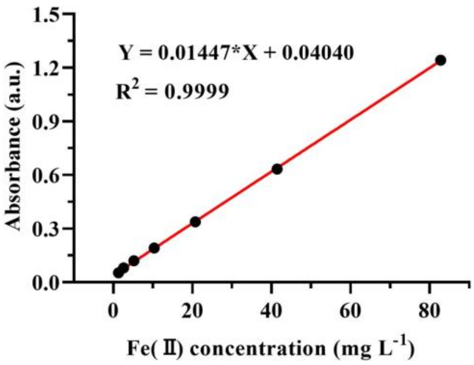

Figure S9. The standard curve of the Fe(II) ions (n = 3, mean ± SD).

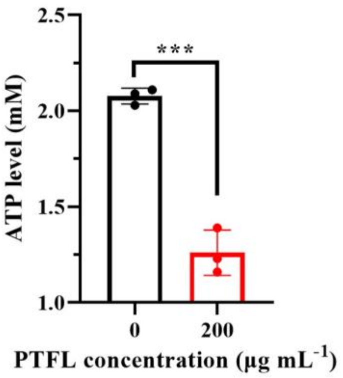

Figure S10. ATP depletion with different PTFL concentrations (n = 3, mean ± SD). Statistical significance was analyzed by Student's t-test (****P* < 0.001)

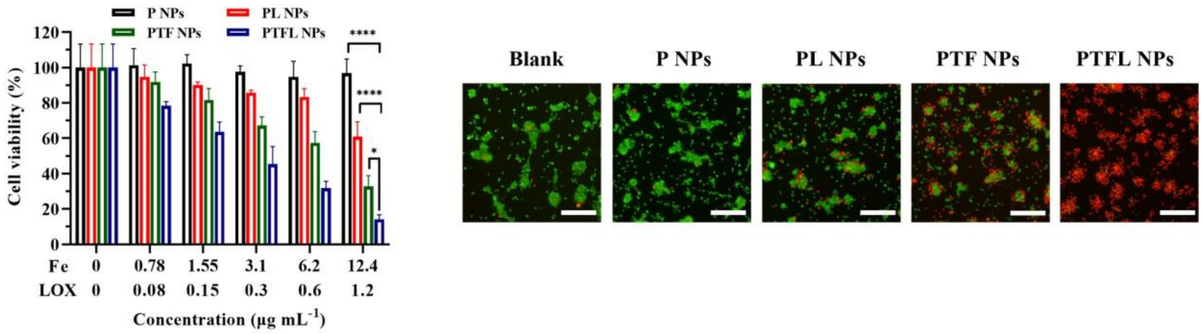

Figure S11. a) Cell viability of CT26 cells after treated with various concentrations of P NPs, PL NPs, PTF NPs, and PTFL NPs for 48 h (n = 3, mean ± SD). b) Confocal fluorescence images of calcein-AM/PI stained CT26 cells after various treatments (PTF NP, PTFL NPs: 72 μg mL^{-1}). Scale bars: 200 μm. (n = 3, mean ± SD). Statistical significance was analyzed by one-way ANOVA (*P < 0.05 and ****P < 0.0001).

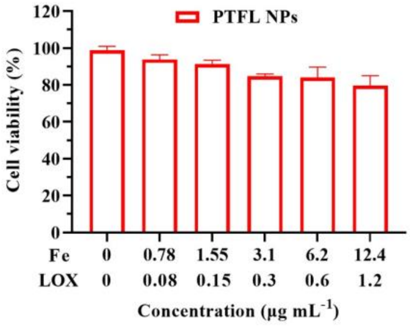

Figure S12. Cell viability of MCF-10A treated with PTFL NPs for 48 h (n = 3, mean ± SD).

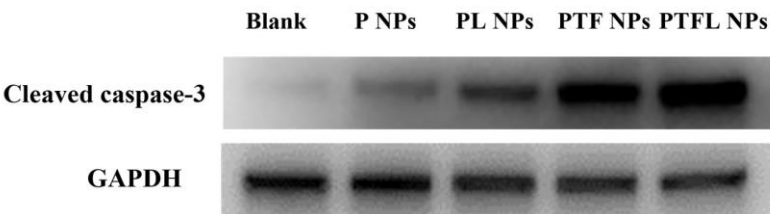

Figure S13. Western blot analysis of the expression levels of cleaved caspase-3 in 4T1 cells after various treatments.

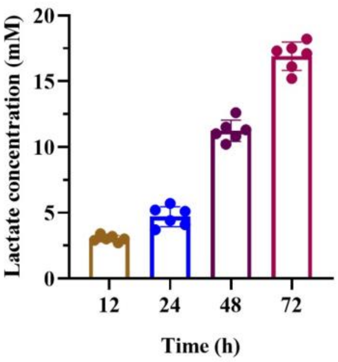

Figure S14. Lactate accumulation in the supernatant of 4T1 cells at different time intervals (n = 6, mean ± SD).

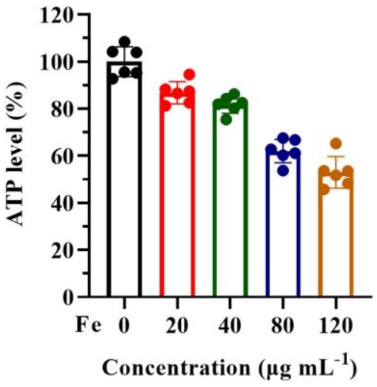

Figure S15. Intracellular ATP level of 4T1 cells treated with various concentrations of PTF NPs (n = 6, mean ± SD).

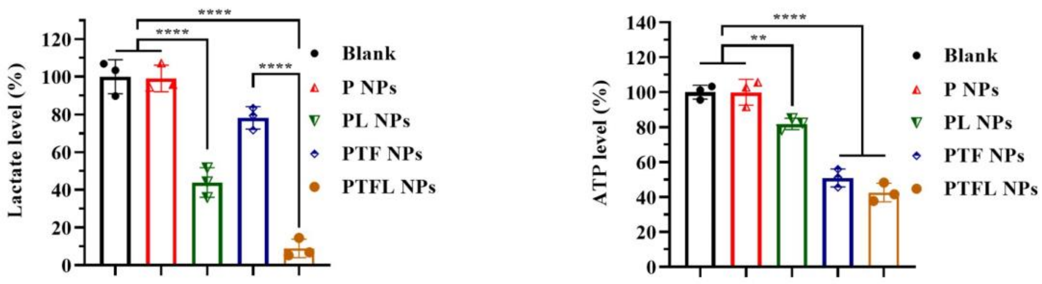

Figure S16. Lactate level in culture medium and intracellular ATP level of CT26 cells after various treatment in b) for 48 h (n = 3, mean ± SD). Statistical significance was analyzed by one-way ANOVA (**$P < 0.01$ and ****$P < 0.0001$).

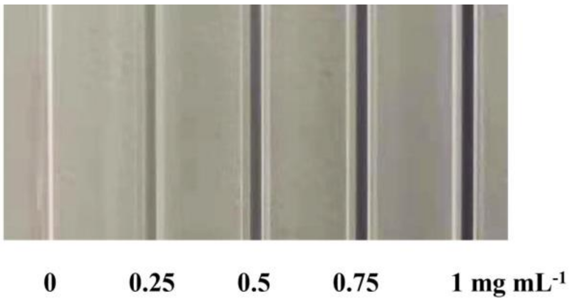

Figure S17. Tissue-mimic phantom for *in vitro* PA imaging of PTFL NPs with various concentrations.

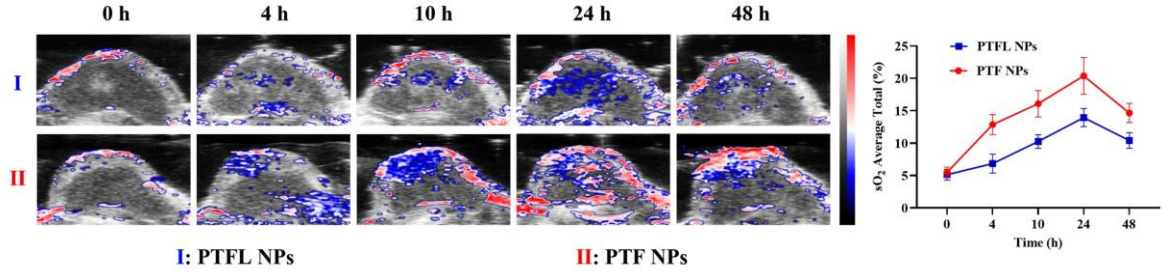

Figure S18. *In vivo* sO_2 images of main tumor sections at different time intervals after i.v. injection of PTF NPs and PTFL NPs and the corresponding quantified intensities of sO_2 average total of tumor areas (n = 3, mean ± SD).

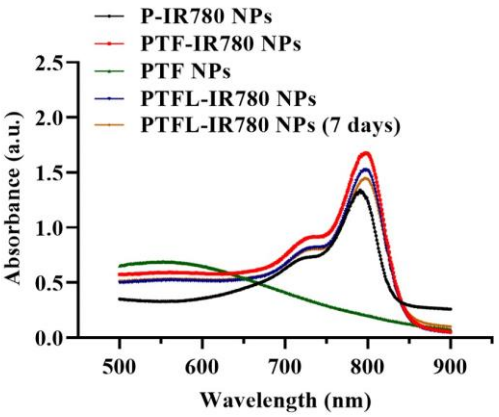

Figure S19. Vis–NIR extinction of P–IR780 NPs, PTF–IR780NPs, PTF NPs, PTFL–IR780 NPs, and PTFL–IR780 NPs after dispersed in PBS for 7 days.

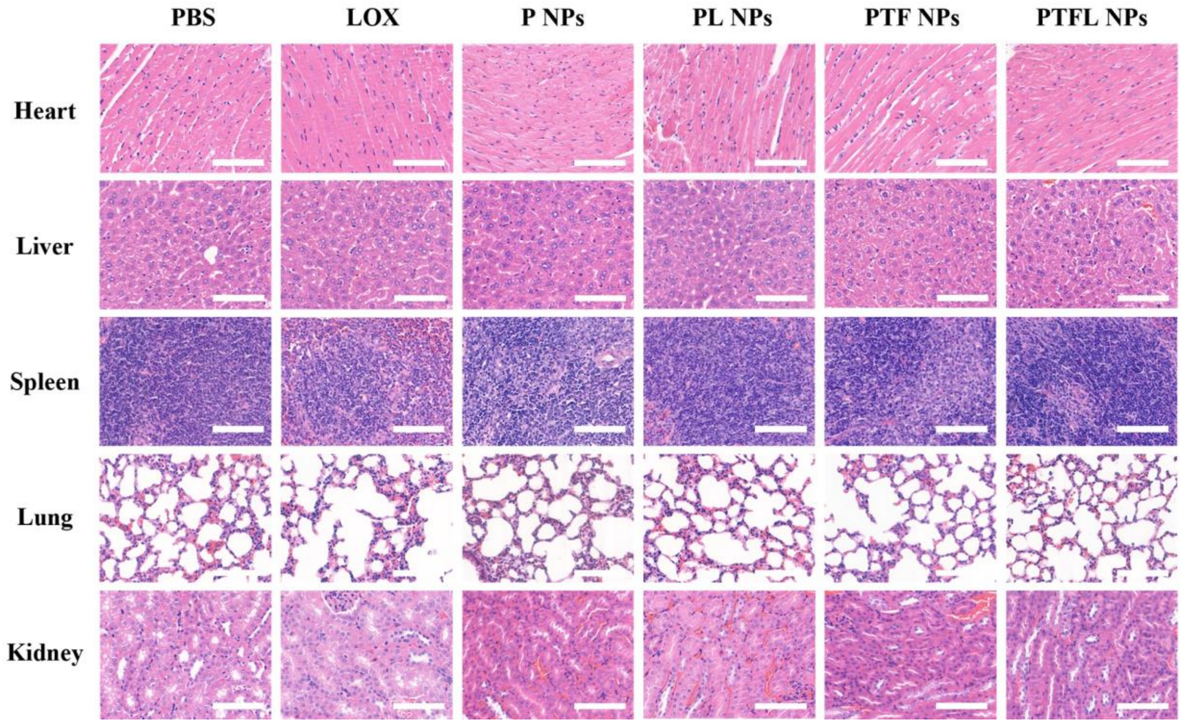

Figure S20. H&E staining images of major organs after various treatments for 16 days. Scale bars: 100 μm.

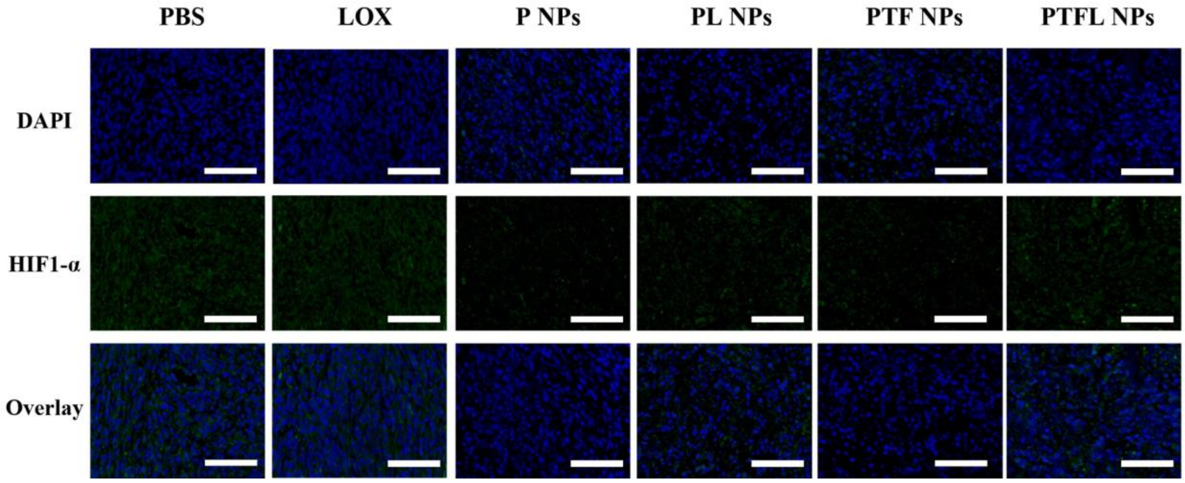

Figure S21. HIF1-α immunofluorescence staining (Green) images after various treatments. Scale bars: 100 μm.
